# Validity and Reliability of Kinovea^®^ for Pelvic Kinematic Measurement in Standing Position and in Sitting Position with 45° of Hip Flexion

**DOI:** 10.3390/s25010250

**Published:** 2025-01-04

**Authors:** Lucía Vicente-Pina, Rocío Sánchez-Rodríguez, Loreto Ferrández-Laliena, Jose Heredia-Jimenez, Julián Müller-Thyssen-Uriarte, Sofía Monti-Ballano, César Hidalgo-García, José Miguel Tricás-Moreno, María Orosia Lucha-López

**Affiliations:** 1 Unidad de Investigación en Fisioterapia, Spin off Centro Clínico OMT-E Fisioterapia SLP, Universidad de Zaragoza, Domingo Miral s/n, 50009 Zaragoza, Spain; l.vicente@unizar.es (L.V.-P.); lferrandez@unizar.es (L.F.-L.); 732751@unizar.es (J.M.-T.-U.); smonti@unizar.es (S.M.-B.); jmtricas@unizar.es (J.M.T.-M.); orolucha@unizar.es (M.O.L.-L.); 2 Department of Physical Education and Sports, Faculty of Education, Economy & Technology, University of Granada, 51001 Ceuta, Spain; herediaj@ugr.es

**Keywords:** range of movement, pelvis, validity, reliability

## Abstract

The anatomy of the pelvis may obscure differences in pelvic tilt, potentially underestimating its correlation with clinical measures. Measuring the total sagittal range of pelvic movement can serve as a reliable indicator of pelvic function. This study assessed the inter- and intra-examiner reliability of the Kinovea^®^ version 0.9.5 and its agreement with the Qualisys System (3D motion capture) for measuring the total pelvic range of movement (ROM) in the sagittal plane, establishing Kinovea^®^’s validity in standing and sitting positions with 45° of hip flexion. A cross-sectional study was conducted with 13 asymptomatic participants. Pelvic kinematics were recorded using both systems. Pelvic posture, anterior and posterior tilt, and total pelvic ROM in the sagittal plane were analyzed. The Intraclass Correlation Coefficient (ICC) was used to evaluate reliability and validity. Additionally, the technical error of measurement (TEM), relative TEM, standard error of measurement, and minimal detectable change (MDC) were calculated to establish Kinovea^®^’s accuracy. Kinovea^®^ demonstrated excellent inter- and intra-examiner reliability for total pelvic ROM in standing and sitting measurements (ICC > 0.90), with relative TEM values below 10% and MDC values between 1.60°and 11.20°. Validity showed good-to-excellent ICC values when comparing Kinovea^®^ and the Qualisys System. This finding suggests that Kinovea^®^ is a valid tool for obtaining reproducible measurements of total pelvic ROM in the sagittal plane in standing and sitting positions, demonstrating excellent-to-good inter- and intra-examiner reliability for pelvic kinematics.

## 1. Introduction

Pelvic tilt is one of the parameters that defines the sagittal orientation of the pelvis in a standing position [1,2]. This posture involves the combination of muscle and ligament forces and pelvic morphology [3]. Spinal curvature, pelvic tilt, and hip range of movement (ROM) act synergistically to maintain verticality [1]. Thus, our body compensates sagittal imbalances through adaptations in these anatomical areas to preserve the postural economy. Pelvic adaptations in the sagittal plane are achieved through the anterior rotation of the pelvis and the posterior rotation of the pelvis. This rotation movement performed around the femoral head’s axis is one of the best mechanisms of regulation of sagittal imbalances and alters lumbar and hip position [4,5]. In physical examinations, pelvic tilt is defined as the angle between the line joining the anterior superior iliac spine and the posterior superior iliac spine and the horizontal line in a static standing position [6,7,8]. Nevertheless, variability in pelvic anatomy may mask differences in pelvic tilt and cause examiners to underestimate any correlation between tilt and other clinical measures [3]. To solve this limitation, the measure of pelvis total ROM in the sagittal plane could be a good estimator of pelvis function, and has the potential to give us information about the capacity of the pelvis to preserve sagittal balance.

Musculoskeletal factors that could be influencing pelvic tilt and lumbopelvic range of motion in standing have been investigated [9,10]. Hip extension deficit appears to be the primary factor influencing sagittal pelvic position and, consequently, spinal curvature [4]. Non-modifiable osseous factors, such as femoral torsion abnormalities, decreased ischiofemoral space, or posterior acetabular wall overcoverage, have the potential to limit hip extension [11]. However, other modifiable musculoskeletal risk factors, including hip flexor tightness, may also play a significant role in reducing hip extension [11].

Hip flexor muscles, due to their anatomical disposition, may have the strongest association with these factors. This muscle group acts by increasing anterior rotation of the ilium when the femur is fixed [6]. It has been found that people with low-back pain have less hip flexor extensibility and greater anterior rotation of the pelvis while walking [12]. Additionally, some studies have shown that anterior rotation of the pelvis is reduced following interventions to increase hip flexor length [7,13].

In physical examinations of sagittal pelvic range of motion in a standing position, the hip flexor muscles may approach their maximal extensibility in people with hip flexor restriction, hindering the active function of the muscles that perform posterior pelvic rotation. Thus, the shortening of these muscles has been associated with altered gluteal activation [14], with the glute muscles being some of the primary pelvic posterior rotation muscles [6]. Then, hip flexor hypomobility could reduce sagittal active pelvic range of motion.

To address this limitation and better approximate sagittal pelvic range of motion, it seems appropriate to use a sitting position, which limits the influence of hip extension mobility on posterior pelvic tilt. Assessments in a sitting position are typically conducted on a surface that induces the hip to 90° of flexion [15,16]. Nonetheless, pelvic motion interacts with lumbar and hip motion and sitting positions at 90° of hip flexion may result in posterior pelvic tilt and lumbar flexion compensation. In fact, pelvis and lumbar compensation starts approximately at 78.4° of hip flexion. Consequently, a sitting position with the femur at 90° with respect to vertical would imply a posterior pelvic tilt that allows for sufficient flexion for this sitting position [15]. For that reason, a traditional sitting position is unsuitable for analyzing total sagittal pelvic range of motion.

Three-dimensional motion analysis [17] or radiographic methods [18,19] in standing and sitting positions have been used to analyze sagittal pelvic position and total ROM. However, these resources are usually not available in clinical settings. Few low-cost instruments have been used to analyze pelvic posture [20,21], and only the Palpation Meter device has been validated [21]. The Palpation Meter device includes a caliper and an inclinometer, allowing us to measure the inclination between the two points where the extremities of the caliper are located. Two-dimensional motion analysis in the sagittal plane has become a cost-efficient resource for motion analysis in different anatomical areas such as the knee [22] or the ankle [23]. Nevertheless, limited studies have analyzed its utility in measuring pelvic kinematics. Recently, Widhalm K et al. assessed the validity of a video-based 2D system against a 3D reference standard in gait analysis. They obtained weak agreement in terms of sagittal pelvic movement due to the differences in the perspective of pelvic markers between 2D and 3D [24].

To our knowledge, no study has validated a 2D motion analysis of total pelvic ROM in a standing position and in a sitting position with 45° of hip flexion.

The purpose of this study was to evaluate the agreement of a 2D motion analysis methodology with a 3D motion capture system in measuring sagittal pelvic ROM in both standing and sitting positions. The second aim was to assess the inter- and intra-examiner reliability of this 2D sagittal pelvic ROM analysis in sitting positions with 45° of hip flexion and standing positions in healthy subjects.

## 2. Materials and Methods

### 2.1. Study Type and Methods

The study consisted of a cross-sectional validity and reliability study. The study was conducted at the Hubema LAB, University of Granada, Ceuta campus (Ceuta Spain).

The study protocol was approved by The Research Ethics Committee of Community of Aragón (code C.I. PI24/303) and adhered to the principles of the Declaration of Helsinki.

### 2.2. Participants

Inclusion criteria were age over eighteen and under seventy and the absence of low-back pain at the time of evaluation. All participants provided written informed consent prior to their inclusion in the study.

Participants were excluded if they had undergone medical infiltration or physiotherapy treatment in the lumbopelvic area within the past three months, or if they had a surgery in the lumbopelvic region.

### 2.3. Instrumentations

Pelvic kinematics were analyzed by using a 3D motion capture system (Qualisys AB, Göteborg, Sweden) which was used as the gold-standard to calculate the agreement with Kinovea^®^ version 0.9.5 (https://www.kinovea.org/download.html, accessed on 10 October 2024). Qualisys’ commitment to quality is solidified by the ISO 9001:2015 certification, European Medical Device Regulation compliance, FDA clearance, and its reputation as a complete, integrative solution provider in motion capture technology (https://www.qualisys.com/life-sciences/human-biomechanics-research/, accessed on 1 May 2024). Previous research has validated Kinovea^®^ across different movements using various 3D motion systems, including the VICON^®^ system [25]. The system consists of twelve infrared high-speed cameras at a rate of 250 Hz. The space was calibrated with a 751.1 mm wand before data collection, ensuring that the wand’s length measurements had standard deviations of less than 0.5 mm. Visual3D (V3D) software version 2024.11.2 (C-Motion Inc., Germantown, MD, USA) was used to analyze sagittal pelvic ROM in a standing position and in a sitting position with 45° of hip flexion.

One of the twelve cameras in the 3D motion capture system was positioned 3 m laterally and perpendicular to the participant at a height of 1 m above the floor. The videos recorded by this camera were analyzed with Kinovea^®^. The camera was adjusted and fixed to be perfectly perpendicular to the pelvis segment. The camera height and distance were selected to account for potential variability among different subjects, thereby minimizing perspective distortion. Since the camera was part of the recording setup of the 3D Qualisys system, any movement of the camera would have required recalibration. Therefore, subjects were always placed in the same recording area, ensuring perpendicular orientation to the camera. Additionally, the required lighting conditions included ambient light that did not create shadowed areas, as such shadows could hinder the visibility of the markers (https://www.kinovea.org/help/en/, accessed on 1 May 2024).

### 2.4. Procedures

Each evaluation protocol was conducted in one session. Prior to the measurement procedure, sociodemographic data including age, sex, weight, and height were collected. Before this, each participant was informed about the evaluation protocol and performed a short warm up consisting of the learning of the pelvic movement in both positions. Measurements were conducted once participants were able to perform anterior and posterior pelvic rotation without compensations in adjacent regions.

The evaluation protocol included the evaluation of sagittal pelvic ROM in a standing and in a sitting position with 45° of hip flexion. This was conducted for the right and left side of the participants. A total of four measurements were performed with randomization of the starting pelvic side and the starting position. Randomization was conducted before each evaluation using RANDOM.ORG (https://www.random.org/, accessed on 10 October 2024) and introducing the four possible measurements (sitting recording from the right side, sitting recording from the left side, standing recording from the right side, and standing recording from the left side).

The 3D motion capture analysis was performed by marking the pelvis and lower extremities and without marking the head, trunk, upper extremities, and feet. Reflective markers were placed with adhesive tape on the participants skin on both lower limbs and pelvis. Anatomical references were used to place the reflective markers. The locations were the anterior and posterior superior iliac spine, the lateral and medial femur epicondyles, and the medial and lateral malleolus. The marker setup followed the CODA protocol recommendations (Charnwood Dynamics Ltd., Leicestershire, UK) for pelvis segment modeling. In addition, two clusters with four markers were fixed at the front part of the thigh and shank on both sides.

To measure sagittal pelvic ROM when standing, participants were instructed to adopt a comfortable stance with their feet positioned hip-width apart, looking straight ahead, and with their arms crossed in front of their chest for a duration of three seconds [25]. The participants were then instructed to perform three repetitions of maximal anterior and posterior pelvic tilts, with each maximal position held for two seconds.

**Figure 1:**
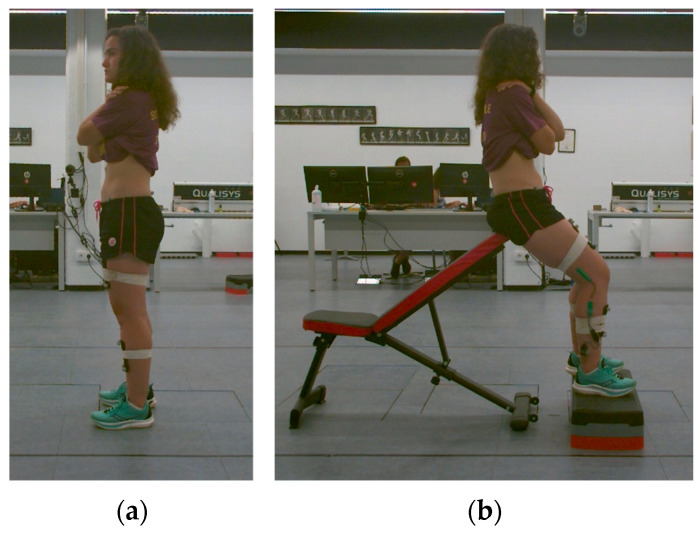
Starting positions for measurement of sagittal pelvic ROM: (**a**) initial position for left-side standing posture; (**b**) initial position for right-side sitting posture with 45° of hip flexion.

During the measurement in the sitting position with 45° of hip flexion, the participants were instructed to sit on their ischial tuberosities, with their legs aligned vertically and their feet resting on the floor. Hip flexion was assessed by an examiner using a two-arm goniometer, and steps were employed to adjust the position (Figure 1). The participants maintained the initial position for three seconds before performing three maximal anterior and posterior pelvic tilts.

Between the standing and sitting measurements, the participants took a brief walk around the room before returning to the evaluation area.

### 2.5. Outcome Variables

For the 2D Kinovea^®^ system analysis, the “angle” tool was used to acquire the sagittal pelvic kinematics. The video recorded by the perpendicular camera was analyzed. The angle calculated was the angle between the line joining the anterior superior iliac spine and the posterior superior iliac spine and the horizontal reference line. Manual detection was used to establish the reference points at the midpoints of the markers

Static pelvic tilt was calculated for the first three seconds of the video. Positive values (>0) referred to anterior pelvic tilt, and negative values (<0) referred to posterior pelvic tilt.

**Figure 2:**
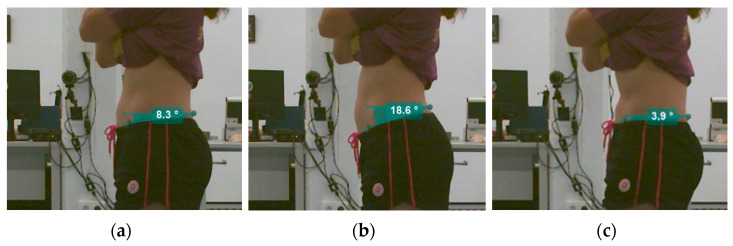
Kinovea^®^ sagittal pelvic kinematics analysis: (**a**) static pelvic tilt; (**b**) maximal anterior rotation of pelvis; (**c**) maximal posterior rotation of pelvis.

Total sagittal pelvic ROM was determined by summing the absolute values of the average angles recorded during maximal anterior and posterior tilt of the pelvis across three repetitions (Figure 2).

Additionally, anterior pelvic tilt was calculated as the absolute value of the difference between the static pelvic posture and the final angle at maximal anterior tilt. Similarly, posterior pelvic tilt was calculated as the absolute value of the difference between the static posture angle and the final angle at maximal posterior tilt.

To process the data obtained with the 3D Qualisys System, Visual3D software was utilized and the total sagittal pelvic ROM was calculated. The proximal references for each pelvic side were defined at the anterior and posterior superior iliac spine, and the distal reference was located at the hip joint center. To obtain the total sagittal pelvic ROM, the minimum (maximal anterior tilt), maximum (maximal posterior tilt), and total ROM (the difference between maximum and minimum) in degrees were computed for each pelvic tilt event and for both sides.

To assess the validity and inter- and intra-examiner reliability of the data obtained from the 2D Kinovea^®^ system and the 3D Qualisys system, two examiners participated in the study. All examiners were blinded to the results recorded by the others. Examiner 1 conducted two recordings with a one-week interval between the first and second sessions to evaluate intra-examiner reliability. Examiner 2 performed an additional recording to assess inter-examiner reliability. Both examiners received standardized instructions: Static pelvic measurements should be taken during the first three seconds. Measurements at the end of the movement were to be taken from the frame immediately before the examiner perceived a change in rotation direction. Final measurements were calculated as the average value from the three movements recorded in each direction.

Finally, for the validity study, the data coming from the 2D Kinovea^®^ system and the 3D Qualisys System was compared.

### 2.6. Sample Size Calculation

Sample size was calculated based on Fernandez et al.’s [26] study on Kinovea^®^ and 3D Motion System reliability using the Granmo calculator (https://www.datarus.eu/aplicaciones/granmo/, accessed on 1 May 2024). Accepting an alpha risk of 0.05 and a power of 0.8 in a two-tailed test of 13 subjects was found to be necessary to find statistical significance, with an Intraclass Correlation Coefficient (ICC) of 0.88 between 2 observers. The ICC null hypothesis was assumed to be 0.5, which indicated moderate reliability [27]. No attrition was expected as it was only 1 measurement session.

### 2.7. Statistical Analysis

Data analysis was performed using SPSS software v.25 (SPSS Inc., Chicago, IL, USA). The level of statistical significance was set at *p* < 0.05.

The distribution of the raw data was assessed using the Kolmogorov–Smirnov test, confirming a normal distribution of all data (*p* > 0.05). Descriptive statistics including means and standard deviations (SDs) were calculated for all of the data. Additionally, minimums and maximums were calculated for total ROM outcomes.

The agreement between the 2D Kinovea^®^ system and the 3D Qualisys System for total sagittal pelvic ROM in both sitting and standing positions was assessed using the ICC, specifically employing a two-way random-effects model with absolute agreement and average measures.

Inter-examiner reliability for the 2D Kinovea^®^ system regarding static pelvic tilt, anterior and posterior pelvic tilt, and total sagittal pelvic ROM was evaluated using the ICC, specifically employing a two-way mixed-effects model with absolute agreement and average measures. Intra-examiner reliability for the same parameters was calculated using the ICC with a two-way random-effects model, also focusing on absolute agreement and average measures. The specific application process was conducted in SPSS using the following path: Analyze–Scale–Reliability Analysis–Statistics–Intraclass Correlation Coefficient–Two-Way Random-Effects Model–Absolute Agreement. This model was specifically chosen because it accounts for variability between subjects as well as measurement deviations between examiners. By considering both as random effects, the model assumes that the sample represents a random population of subjects and inspectors, thereby enhancing the generalizability of the results.

To interpret the ICC values, this study adopted the following criteria: >0.90 indicates excellent reliability, 0.75–0.90 indicates good reliability, 0.50–0.75 indicates moderate reliability, and <0.50 indicates poor reliability [27].

The accuracy obtained with the 2D Kinovea^®^ system was assessed using both the absolute and relative technical error of measurement (TEM and rTEM), the standard error of measurement (SEM), and the minimal detectable change (MDC).

The tEM was calculated using the formula $TEM=\surd \frac{\sum ({X_{1}-X_{2})}^{2}}{2N}$, where X_1_ and X_2_ represent each participant’s measurement and N represents the number of subjects; the rTEM was calculated as the percentage that the TEM represents in relation to the average of the measurements [28]; the SEM was calculated as $SEM=SD\sqrt{1-ICC}$; and finally, the MDC was calculated as $MDC=1.96\times SEM\times \sqrt{2}$.

## 3. Results

The study group consisted of 13 subjects (5 women/8 men; 39.15 ± 10.61 years; IMC 24.56 ± 2.38) without low-back pain.

The descriptive analysis of pelvic ROM values obtained by examiner 1 with the 2D Kinovea^®^ system and the data from the intra-examiner reliability analysis are shown in Table 1. The intra-examiner reliability showed excellent correlations (ICC > 0.90) for all measurements. TEM values in a standing position ranged between 0.3° and 0.6° with an rTEM of 1.8% to 7.5%. Higher values were found in a sitting position with 45° of hip flexion, where TEM values ranged from 0.3° to 1.6° with rTEM values of 1.9% to −14.0%. The higher MDC (11.2°) was found for the right-side total sagittal pelvic ROM in a sitting position with 45° of hip flexion.

Table 2 shows the descriptive analysis of pelvic ROM values obtained by examiner 1 and 2 with the 2D Kinovea^®^ system and the inter-examiner reliability study. The inter-examiner reliability of all of the variables found excellent correlations (ICC > 90), except for left-side posterior pelvic tilt in a sitting position with 45° of hip flexion which showed good reliability (ICC = 0.88). Left-side anterior pelvic tilt in a sitting position with 45° of hip flexion showed the worst accuracy values (rTEM = 12.6%; MDC: 10.0°).

The reliability of the 2D Kinovea^®^ system in measuring total sagittal pelvic ROM when compared to the 3D Qualisys System was found to be excellent (ICC > 90) in all of the records except for left-side total sagittal pelvic ROM in a standing position (ICC = 0.88) Descriptive statistics with the minimum and maximum values of total sagittal pelvic ROM obtained by the 2D Kinovea^®^ system and the 3D Qualisys System and the reliability analysis are shown in Table 3.

## 4. Discussion

This study provides insights into the validity and reliability of a video-based system for assessing pelvic sagittal ROM parameters. Considering the agreement between the 2D Kinovea^®^ system and the 3D Qualisys System values, excellent-to-good correlations were found for total sagittal pelvic ROM. Our second aim was to evaluate the inter- and intra-examiner reliability, and the results indicated that the reliability ranged from good to excellent, with worse accuracy in the sitting position.

With regard to intra-examiner reliability with the 2D Kinovea^®^ system for pelvic ROM outcomes, we report excellent ICCs across all measurements. Our findings are broadly consistent with those of other studies employing different devices to measure pelvic tilt. For instance, Prushansky et al. [20] in their study on digital inclinometry for quantifying sagittal pelvic kinematics in the standing position reported excellent reliability for anterior and posterior pelvic tilt as well as total pelvic ROM in women (ICC = 0.90–0.94; SEM: 2.0–2.3°), although their reliability values ranged from excellent to moderate in men (ICC = 0.93–0.60; SEM: 1.7–2.1°). Similarly, Barbosa et al. [29], using a methodology comparable to ours and employing photographs to analyze static pelvic tilt in a standing position one week apart, also reported excellent results (ICC = 0.96–0.95) consistent with studies using a caliper-based inclinometer [21,30].

In terms of inter-examiner reliability, Barbosa et al. [29], in their assessment of static pelvic tilt, reported excellent ICC values (0.96–0.95), while a slightly lower reliability was observed with the Palpation Meter (PALM) device (ICC = 0.89; SEM: 3.7°) [21]. Other studies analyzing the inter-examiner reliability of the 2D Kinovea^®^ system for recording joint angles during walking have reported excellent ICCs for sagittal angles of the hip, knee, and ankle joints (ICC = 0.96–0.98) [26]. These findings align with those of our study, which demonstrated excellent ICC values for most of the measurements, except for left-side posterior pelvic tilt in the sitting position with 45° of hip flexion, where reliability was rated as good (ICC = 0.88).

However, despite the significant and excellent or good ICC values observed for inter- and intra-examiner reliability, wide confidence intervals were reported for some variables, indicating moderate correlation in certain cases. This was particularly evident in the anterior and posterior pelvic tilt measurements during the sitting position, likely due to the greater range of pelvic movement and the increased difficulty in precisely identifying markers in this posture compared to the standing position. Implementing strategies such as using a black background or color-coded markers or adjusting the height and positioning of the video camera could enhance measurement accuracy.

Additionally, the TEM and rTEM values reflect lower accuracy in the sitting position. However, no reference values of the TEM had been established previously in this region. Taking anthropometry as a reference, rTEM values of 10% are accepted [31], and in this context, only sitting measurements would fail to achieve an acceptable rTEM. This is consistent with the higher MDC values observed in the sitting position. Previous studies assessing muscle flexibility using a digital motion analysis in 2D report rTEM values ranging from 2.6% to 12.4%, which are similar to our results and have been deemed acceptable [32]. rTEM values are dependent on each position and assessment, and the observed differences may be attributed to variability in standardization. Our study obtained lower values of accuracy for static pelvic tilt in the sitting position, which could be improved by providing better support for the sitting posture and using a standardized frame for pelvic analysis, as small movements to adjust posture typically occur within the first minutes of sitting [33].

The validity study of pelvic ROM measured with the 2D Kinovea^®^ system compared to the 3D Qualisys System has shown excellent (ICC > 0.90) to good (ICC 0.90–0.75) results for all ROM measurements and found no significant differences between the two systems. However, previous studies comparing the agreement between 3D motion analysis systems and the 2D Kinovea^®^ system have reported discrepancies between them [26,34]. A possible explanation for the better result in this study could be that other studies analyzed participants while walking, rather than in a standing or sitting position. Our tasks focused on isolated pelvic kinematics, measuring larger angles that represent total pelvic ROM, rather than small pelvic kinematics between strides. These relatively static postures favor the evaluation of isolated pelvic ROM and provide us with insights into the active pelvic adjustments that an individual can make. Despite these findings, we must consider that both left-side tests exhibited wide confidence intervals, with ICC values indicating moderate correlation. This may be due to the greater sensitivity of the 3D Qualisys System to small leg movements, which could potentially alter pelvic kinematics [35], as the 3D Qualisys System uses the hip joint center as the reference for pelvic kinematics, while the 2D Kinovea^®^ system uses the horizontal plane as its reference.

Considering total pelvic ROM in the standing position of both the 2D Kinovea^®^ system (18.9° on right and 16.9° on left side) and the 3D Qualisys System (18.8° on right and 16.3° on left side), these values are similar to the total pelvic ROM found by Prushansky et al. [20] in women (19.8–18.0°) but slightly larger than those in men (12.5–14.6°). In contrast, both are lower than the values found by Van Goeverden et al. [36] in injured (21.1°) and non-injured (27.2°) athletes. Nonetheless, in Van Goeverden et al.’s study, participants performed pelvic tilt to recreate a step movement, with one leg in a midstance position and with the trailing swing leg in a high knee position, avoiding rectus femoris tension and thus allowing for more pelvic movement.

In relation to total pelvic total ROM in the sitting position, no studies have been found that specifically analyze the sitting position with 45° of hip flexion. The most comparable study, conducted by Swärd et al. [37], analyzed total pelvic ROM while sitting with 90° of hip and knee flexion and found values of 22.4° in healthy subjects and 21.1° in participants with hip pathology. Considering that age is a determining factor for reductions in ROM [38,39,40], our study shows similar or even greater ROM in an older sample (mean age of forty years old) compared to the 17–25-year-old participants in other studies [36,37], confirming a sitting position with 45° of hip flexion as a more accurate position for measuring total pelvic ROM.

Regarding anterior and posterior pelvic tilt in the standing position, our data reveal a similar ROM in both directions, with a slight difference of 0.2° to 3° to anterior pelvic tilt. These data are consistent with the literature, though some studies found no difference [36] and others found a difference of 4° to 6° to anterior tilt [6,20]. If we examine sitting positions, we can find similar results, with a tendency toward more anterior ROM, which also aligns with the literature [37].

Finally, if we analyze static pelvic tilt in a standing position, previous research describes heterogeneous data with values ranging from 26° to 7.9° [7,29,30,41]. Our mean data show smaller values of anterior rotation of the pelvis than those proposed by Krawezky et al. [8] as the reference values for pelvic tilt in a standing position were established at 12.26° and higher than those obtained by using the PALM device before and after hip flexor stretching [7]. Nevertheless, according to Preece et al. [3], differences in static pelvic tilt should be at least 4° because of the variability in pelvic morphology, and thus, the difference found in our sample could be due to anatomic variations.

### Study Limitations

For this study, the following points should be considered limitations. Firstly, the relatively small sample size only includes people with a normal BMI, and therefore, we are unable to consider the effect of abdominal fat tissue on the measurements. Second, although we conducted a validation study, the true gold standard would be the radiographic method [4]. However, with the radiographic method, pelvic tilt is measured using different anatomical references, making the values non-comparable. Additionally, using X-rays is not innocuous for asymptomatic individuals.

Thirdly, we selected an asymptomatic population to minimize movement alterations. However, in symptomatic populations, where a reduced range of motion is more likely, the accuracy values may be deteriorated.

Finally, the reliability study measurements could have been registered on different days, since fluctuation in localization of the anatomical references may be an important source of variability [28]. However, in this case, activities between evaluation sessions on different days such as sitting time or physical activities could influence the measurements.

## 5. Conclusions

The results of this study suggest that the 2D Kinovea^®^ system is a valid tool which can be used to obtain reproducible measurements of total pelvic ROM in the sagittal plane in a standing position and in a sitting position with 45° of hip flexion.

The 2D Kinovea^®^ system has shown excellent-to-good inter- and intra-examiner reliability for the measurement of pelvic kinematics. It seems that the 2D Kinovea^®^ system may be a good, low-cost alternative for the analysis of pelvic sagittal movement in a standing position and in a sitting position with 45° of hip flexion.

## Figures and Tables

**Table 1:** Descriptive analysis of pelvic ROM values obtained with the 2D Kinovea^®^ system and intra-examiner reliability analysis.

	Examiner 1, 1st Measurement (Mean ± SD)	Examiner 1, 2nd Measurement (Mean ± SD)	ICC ± [95% CI]	*p*-Value	TEM (°)	rTEM (%)	SEM (°)	MDC (°)
**Standing position**								
**Right side**								
Static pelvic tilt (°)	9.4° ± 2.9°	8.6° ± 3.8°	0.99 [0.95–1.00]	<0.01	0.5°	5.2	0.9	2.6
Anterior pelvic tilt (°)	9.7° ± 5.0°	9.7° ± 5.1°	0.99 [0.97–1.00]	<0.01	0.6°	6.0	0.8	2.3
Posterior pelvic tilt (°)	9.2° ± 2.9°	9.2° ± 2.8°	0.99 [0.94–1.00]	<0.01	0.5°	5.3	1.1	3.1
Total sagittal pelvic ROM (°)	18.9° ± 6.7°	18.9° ± 6.7°	1.00 [1.00–1.00]	<0.01	0.3°	1.8	0.6	1.7
**Left side**								
Static pelvic tilt (°)	9.5° ± 3.5°	9.7° ± 3.4°	0.99 [0.98–1.00]	<0.01	0.4°	4.0	0.7	2.1
Anterior pelvic tilt (°)	10.3° ± 4.1°	9.9° ± 3.5°	0.99 [0.97–1.00]	<0.01	0.5°	4.7	0.9	2.5
Posterior pelvic tilt (°)	7.1° ± 2.8°	7.3° ± 3.3°	0.98 [0.93–1.00]	<0.01	0.5°	7.5	0.9	2.6
Total sagittal pelvic ROM (°)	16.8° ± 5.3°	17.8° ± 5.6°	1.00 [0.99–1.00]	<0.01	0.5°	2.6	0.9	2.4
**Sitting position with 45° of hip flexion**								
**Right side**								
Static pelvic tilt (°)	−4.9° ± 7.7°	−4.7° ± 7.6°	1.00 [0.99–1.00]	<0.01	0.5°	−11.4	0.3	0.7
Anterior pelvic tilt (°)	15.0° ± 4.4°	15.3° ± 4.3°	0.95 [0.83–0.99]	<0.01	1.3°	8.5	3.2	9.0
Posterior pelvic tilt (°)	8.2° ± 4.7°	8.2° ± 4.0°	0.96 [0.85–0.99]	<0.01	0.4°	−11.1	1.6	4.6
Total sagittal pelvic ROM (°)	23.2° ± 7.1°	23.5° ± 6.1°	0.97 [0.89–0.99]	<0.01	1.6°	6.8	4.0	11.2
**Left side**								
Static pelvic tilt (°)	−3.1° ± 6.0°	−3.0° ± 5.9°	1.00 [0.99–1.00]	<0.01	0.4°	−14.0	0.2	0.5
Anterior pelvic tilt (°)	13.2° ± 5.1°	13.7° ± 5.0°	0.94 [0.75–0.98]	<0.01	0.3°	1.9	3.4	9.3
Posterior pelvic tilt (°)	10.5° ± 5.5°	9.8° ± 5.7°	0.93 [0.69–0.98]	0.01	0.5°	4.8	2.7	7.4
Total sagittal pelvic ROM (°)	23.6° ± 1.6°	23.5° ± 4.9°	0.99 [0.98–1.00]	<0.01	0.5°	2.0	1.8	4.9

ICC: Intraclass Correlation Coefficient; CI: confidence interval; TEM: technical error of measurement; rTEM: relative technical error of measurement; ROM: range of movement; SEM: standard error of measurement; MDC: minimal detectable change.

**Table 2:** Descriptive analysis of pelvis ROM values obtained with the 2D Kinovea^®^ system and inter-examiner reliability analysis.

	Examiner 1 1st Measurement (Mean ± SD)	Examiner 2 1st Measurement (Mean ± SD)	ICC ± [95% CI]	*p*-Value	TEM (°)	rTEM (%)	SEM (°)	MDC (°)
**Standing position**								
**Right side**								
Static pelvic tilt (°)	9.4° ± 2.9°	8.8° ± 3.9°	0.98 [0.94–1.00]	<0.01	0.7°	7.0	1.1°	3.1°
Anterior pelvic tilt (°)	9.7° ± 5.0°	9.5° ± 4.9°	0.99 [0.96–1.00]	<0.01	0.7°	7.4	1.0°	2.7°
Posterior pelvic tilt (°)	9.2° ± 2.9°	9.2° ± 3.1°	0.97 [0.90–0.99]	<0.01	0.7°	7.3	1.5°	4.1°
Total sagittal pelvic ROM (°)	18.9° ± 6.7°	18.7° ± 6.8°	1.00 [0.99–1.00]	<0.01	0.4°	2.3	0.9°	2.4°
**Left side**								
Static pelvic tilt (°)	9.5° ± 3.5°	9.5° ± 3.5°	1.00 [0.98–1.00]	<0.01	0.3°	3.5	0.6°	1.7°
Anterior pelvic tilt (°)	10.3° ± 4.1°	9.7° ± 3.6°	0.99 [0.96–1.00]	<0.01	0.5°	5.4	3.3°	9.2°
Posterior pelvic tilt (°)	7.1° ± 2.8°	7.1° ± 3.0°	1.00 [0.98–1.00]	<0.01	0.3°	3.6	0.6°	1.5°
Total sagittal pelvic ROM (°)	16.8° ± 5.3°	16.7° ± 5.6°	0.99 [0.97–1.00]	<0.01	0.6°	3.7	3.0°	8.4°
**Sitting position with 45° of hip flexion**								
**Right side**								
Static pelvic tilt (°)	−4.9° ± 7.7°	−4.8° ± 8.0°	1.00 [0.99–1.00]	<0.01	0.5°	−9.8	0.2°	0.6°
Anterior pelvic tilt (°)	15.0° ± 4.4°	14.5° ± 4.4°	0.95 [0.82–0.99]	<0.01	1.4°	9.7	1.0°	2.7°
Posterior pelvic tilt (°)	8.2° ± 4.7°	8.0° ± 5.0°	1.00 [0.98–1.00]	<0.01	0.5°	5.7	0.4°	1.2°
Total sagittal pelvic ROM (°)	23.2° ± 7.1°	22.5° ± 7.2°	0.98 [0.94–1.00]	<0.01	1.3°	5.9	1.4°	3.7°
**Left side**								
Static pelvic tilt (°)	−3.1° ± 6.0°	−4.0° ± 5.3°	0.94 [0.76–0.98]	<0.01	0.4°	−9.7	0.9°	2.5°
Anterior pelvic tilt (°)	13.2° ± 5.1°	13.5° ± 4.1°	0.93 [0.71–0.98]	<0.01	1.7°	12.6	3.6°	10.0°
Posterior pelvic tilt (°)	10.5° ± 5.5°	9.5° ± 3.5°	0.88 [0.52–0.97]	<0.01	0.3°	3.2	3.4°	9.5°
Total sagittal pelvic ROM (°)	23.6° ± 1.6°	22.9° ± 5.1°	0.99 [0.92–1.00]	<0.01	0.8°	3.3	2.6°	7.1°

ICC: Intraclass Correlation Coefficient; CI: confidence interval; TEM: technical error of measurement; rTEM: relative technical error of measurement; ROM: range of movement; SEM: standard error of measurement; MDC: minimal detectable change.

**Table 3:** Descriptive values and validity study of the 2D Kinovea^®^ system and the 3D Qualisys System.

	Examiner 1, 1st Measurement Mean [Min-Max]; ±SD	3D Qualisys System Mean [Min-Max]; ±SD	ICC ± [95% CI]	*p*-Value
	**Standing position**			
Right-side total sagittal pelvic ROM (°)	18.9 [8.7–28.8]; ± 6.7°	18.8 [7.3–33.0]; ± 7.3°	0.97 [0.88–0.99]	<0.01
Left-side total sagittal pelvic ROM (°)	16.8 [8.6–25.4]; ± 5.3°	16.3 [5.4–26.8]; ± 6.3°	0.88 [0.54–0.97]	<0.01
	**Sitting position with 45° of hip flexion**			
Right-side total sagittal pelvic ROM (°)	23.2 [13.3–32.8]; ± 7.1°	25.8 [4.6–42.0]; ± 10.2°	0.97 [0.87–0.99]	0.01
Left-side total sagittal pelvic ROM (°)	23.6 [15.5–30.2]; ± 1.6°	26.8 [10.7–39.9]; ± 7.3°	0.98 [0.93–1.00]	<0.01

ICC: Intraclass Correlation Coefficient; CI: confidence interval; ROM: range of motion.

## Data Availability

The datasets presented in this study are available on request from the corresponding author. All data covered by this study are included in this manuscript.

## References

[1] Pierannunzii L. (2017). Pelvic Posture and Kinematics in Femoroacetabular Impingement: A Systematic Review. J. Orthop. Traumatol..

[2] Shon W.Y., Gupta S., Biswal S., Hur C.Y., Jajodia N., Hong S.J., Myung J.S. (2008). Validation of a Simple Radiographic Method to Determine Variations in Pelvic and Acetabular Cup Sagittal Plane Alignment after Total Hip Arthroplasty. Skeletal Radiol..

[3] Preece S.J., Willand P., Herrington L., Bowker P. (2008). Variation in Pelvic Morphology May Prevent the Identification of Anterior Pelvic Tilt. J. Man. Manip. Ther..

[4] Roussouly P., Pinheiro-Franco J.L. (2011). Biomechanical Analysis of the Spino-Pelvic Organization and Adaptation in Pathology. Eur. Spine J..

[5] Le Huec J.C., Thompson W., Mohsinaly Y., Barrey C., Faundez A. (2019). Sagittal Balance of the Spine. Eur. Spine J..

[6] Takaki S., Kaneoka K., Okubo Y., Otsuka S., Tatsumura M., Shiina I., Miyakawa S. (2016). Analysis of Muscle Activity during Active Pelvic Tilting in Sagittal Plane. Phys. Ther. Res..

[7] Preece S.J., Tan Y.F., Alghamdi T.D.A., Arnall F.A. (2021). Comparison of Pelvic Tilt Before and After Hip Flexor Stretching in Healthy Adults. J. Manip. Physiol. Ther..

[8] Krawczky B., Pacheco A.G., Mainenti M.R.M. (2014). A Systematic Review of the Angular Values Obtained by Computerized Photogrammetry in Sagittal Plane: A Proposal for Reference Values. J. Manip. Physiol. Ther..

[9] Cejudo A., Centenera-Centenera J.M., Santonja-Medina F. (2021). The Potential Role of Hamstring Extensibility on Sagittal Pelvic Tilt, Sagittal Spinal Curves and Recurrent Low Back Pain in Team Sports Players: A Gender Perspective Analysis. Int. J. Environ. Res. Public Health.

[10] Herrera M.C., Amasay T., Egret C. (2021). Lack of Correlation Between Natural Pelvic Tilt Angle with Hip Range of Motion, and Hip Muscle Torque Ratio. Int. J. Exerc. Sci..

[11] Feng R., Hatem M., Nimmons S.J., Disantis A., Martin R.R.L., Martin H.D. (2022). Hip Physical Examination Extension Loss and Radiographic Osseous Findings in Patients with Low Back Pain and Nonarthritic Hips. Bayl. Univ. Med. Cent. Proc..

[12] Jiménez-Del-Barrio S., Mingo-Gómez M.T., Estébanez-De-Miguel E., Saiz-Cantero E., Del-Salvador-Miguélez A.I., Ceballos-Laita L. (2020). Adaptations in Pelvis, Hip and Knee Kinematics during Gait and Muscle Extensibility in Low Back Pain Patients: A Cross-Sectional Study. J. Back. Musculoskelet. Rehabil..

[13] Mendiguchia J., Gonzalez De la Flor A., Mendez-Villanueva A., Morin J.-B., Edouard P., Garrues M.A. (2020). Training-Induced Changes in Anterior Pelvic Tilt: Potential Implications for Hamstring Strain Injuries Management. J. Sports Sci..

[14] Mills M., Frank B., Goto S., Blackburn T., Cates S., Clark M., Aguilar A., Fava N., Padua D. (2015). Effect of Restricted Hip Flexor Muscle Length on Hip Extensor Muscle Activity and Lower Extremity Biomechanics in College-Aged Female Soccer Players. Int. J. Sports Phys. Ther..

[15] Stephens A., Munir S., Shah S., Walter W.L. (2015). The Kinematic Relationship between Sitting and Standing Posture and Pelvic Inclination and Its Significance to Cup Positioning in Total Hip Arthroplasty. Int. Orthop..

[16] Yang Y., Chen H., Zhou Q., Wang B., Zhu Z., Qiu Y., Sun X. (2024). Can Pelvic Incidence Affect Changes in Sagittal Spino-Pelvic Parameters between Standing and Sitting Positions in Individuals with Lumbar Degenerative Disease?. Eur. Spine J..

[17] Mousavi S.J., Tromp R., Swann M.C., White A.P., Anderson D.E. (2018). Between-Session Reliability of Opto-Electronic Motion Capture in Measuring Sagittal Posture and 3-D Ranges of Motion of the Thoracolumbar Spine. J. Biomech..

[18] Ferenczi A., Moraux A., Le Gall F., Thevenon A., Wieczorek V. (2020). Relationship Between Spinal-Pelvic Sagittal Balance and Pelvic-Femoral Injuries in Professional Soccer Players. Orthop. J. Sports Med..

[19] Bailey J.F., Sparrey C.J., Been E., Kramer P.A. (2016). Morphological and Postural Sexual Dimorphism of the Lumbar Spine Facilitates Greater Lordosis in Females. J. Anat..

[20] Prushansky T., Ezra N., Kurse N., Man L., Schneiderman Y. (2008). Reproducibility of Sagittal Pelvic Tilt Measurements in Normal Subjects Using Digital Inclinometry. Gait Posture.

[21] Hagins M., Brown M., Cook C., Gstalder K., Kam M. (1998). Intratester and Intertester Reliability of’the Palpation Meter (PALM) in Measuring Pelvic Position. J. Man. Manip. Ther..

[22] Fadari Dehcheshmeh P., Gandomi F., Maffulli N. (2021). Effect of Lumbopelvic Control on Landing Mechanics and Lower Extremity Muscles’ Activities in Female Professional Athletes: Implications for Injury Prevention. BMC Sports Sci. Med. Rehabil..

[23] Robles-Palazón F.J., Ruiz-Pérez I., Oliver J.L., Ayala F., Sainz de Baranda P. (2021). Reliability, Validity, and Maturation-Related Differences of Frontal and Sagittal Plane Landing Kinematic Measures during Drop Jump and Tuck Jump Screening Tests in Male Youth Soccer Players. Phys. Ther. Sport.

[24] Widhalm K., Durstberger S., Greisberger A., Wolf B., Putz P. (2024). Validity of Assessing Level Walking with the 2D Motion Analysis Software TEMPLO and Reliability of 3D Marker Application. Sci. Rep..

[25] Fernández-González P., Koutsou A., Cuesta-Gómez A., Carratalá-Tejada M., Miangolarra-Page J.C., Molina-Rueda F. (2020). Reliability of Kinovea^®^ Software and Agreement with a Three-Dimensional Motion System for Gait Analysis in Healthy Subjects. Sensors.

[26] Ludwig O., Dindorf C., Kelm S., Kelm J., Fröhlich M. (2024). Muscular Strategies for Correcting the Pelvic Position to Improve Posture—An Exploratory Study. J. Funct. Morphol. Kinesiol..

[27] Koo T.K., Li M.Y. (2016). A Guideline of Selecting and Reporting Intraclass Correlation Coefficients for Reliability Research. J. Chiropr. Med..

[28] Harris E.F., Smith R.N. (2009). Accounting for Measurement Error: A Critical but Often Overlooked Process. Arch. Oral. Biol..

[29] Barbosa A., Bonifácio D., Lopes Í., Martins F., Barbosa M., Vitorino D., Barbosa A. (2013). Intra-and Inter-Rater Reliability in Photogrammetric Pelvic Tilt Angles Analysis. Int. J. Ther. Rehabil..

[30] Beardsley C., Egerton T., Skinner B. (2016). Test-Re-Test Reliability and Inter-Rater Reliability of a Digital Pelvic Inclinometer in Young, Healthy Males and Females. PeerJ.

[31] Perini T.A., Lameira De Oliveira G., Dos J., Ornellas S., Palha De Oliveira F. (2005). Technical Error of Measurement in Anthropometry. Rev. Bras. Med. Esporte.

[32] Fourchet F., Buchheit M., Materne O., Horobeanu C., Hudacek T., Sebo D., Millet G.P. (2013). Reliability of a Novel Procedure to Monitor the FLexibility of Lower Limb Muscle Groups in Highly-Trained Adolescent Athletes. Phys. Ther. Sport..

[33] Davidson J.M., Callaghan J.P. (2025). A Week-Long Field Study of Seated Pelvis and Lumbar Spine Kinematics during Office Work. Appl. Ergon..

[34] Littrell M.E., Chang Y.H., Selgrade B.P. (2018). Development and Assessment of a Low-Cost Clinical Gait Analysis System. J. Appl. Biomech..

[35] Park S.H., Yong M.S., Lee H.Y. (2022). Lower-Limb Kinematic Change during Pelvis Anterior and Posterior Tilt in Double-Limb Support in Healthy Subjects with Knee Malalignment. Int. J. Environ. Res. Public Health.

[36] Van Goeverden W., Langhout R.F.H., Barendrecht M., Tak I.J.R. (2019). Active Pelvic Tilt Is Reduced in Athletes with Groin Injury; a Case-Controlled Study. Phys. Ther. Sport.

[37] Swärd Aminoff A., Agnvall C., Todd C., Jónasson P., Sansone M., Thoreson O., Swärd L., Karlsson J., Baranto A. (2018). The Effect of Pelvic Tilt and Cam on Hip Range of Motion in Young Elite Skiers and Nonathletes. Open Access J. Sports Med..

[38] Cejudo A., Robles-Palazón F.J., Ayala F., De Ste Croix M., Ortega-Toro E., Santonja-Medina F., De Baranda P.S. (2019). Age-Related Differences in Flexibility in Soccer Players 8-19 Years Old. PeerJ.

[39] Sung P.S. (2016). Different Coordination and Flexibility of the Spine and Pelvis during Lateral Bending between Young and Older Adults. Hum. Mov. Sci..

[40] Nakanishi S., Watanabe K., Ouchi K., Hakozaki M., Oi N., Konno S. (2024). Reference Values of Lumbar Spine Range of Motion by Sex and Age Based on the Assessment of Supine Trunk Lateral Bending-A Preliminary Study. Fukushima J. Med. Sci..

[41] Bibrowicz K., Szurmik T., Kurzeja P., Bibrowicz B., Ogrodzka-Ciechanowicz K. (2024). Pelvic Tilt and Stiffness of the Muscles Stabilising the Lumbo-Pelvic-Hip (LPH) Complex in Tensiomyography Examination. PLoS ONE.

